# Tailoring of silica-based nanoporous pod by spermidine multi-activity

**DOI:** 10.1038/s41598-020-77957-4

**Published:** 2020-12-03

**Authors:** Giulia Della Rosa, Riccardo Di Corato, Sara Carpi, Beatrice Polini, Antonietta Taurino, Lorena Tedeschi, Paola Nieri, Rosaria Rinaldi, Alessandra Aloisi

**Affiliations:** 1 Mathematics and Physics “E. De Giorgi” Department, University of Salento, Via Arnesano, 73100 Lecce, Italy; 2 Center for Biomolecular Nanotechnologies (CBN), Istituto Italiano di Tecnologia (IIT), Via Barsanti, Arnesano, 73010 Lecce, Italy; 3 Department of Pharmacy, University of Pisa, Via Bonanno Pisano, 56126 Pisa, Italy; 4 Centro Interdipartimentale di Farmacologia Marina, MARine PHARMA Center, University of Pisa, Via Bonanno Pisano, 56126 Pisa, Italy; 5 Institute for Microelectronics and Microsystems (IMM), CNR, Via Monteroni, 73100 Lecce, Italy; 6 Oligonucleotides Laboratory, Institute of Clinical Physiology (IFC), CNR, Via Moruzzi, 56124 Pisa, Italy; 7 ISUFI, University of Salento, Via Monteroni, 73100 Lecce, Italy; 8 Present Address: Department of Neuroscience and Brain Technologies (NBT), Istituto Italiano di Tecnologia (IIT), Via Morego, 16163 Genova, Italy; 9 Present Address: Institute for Microelectronics and Microsystems (IMM), CNR, Via Monteroni, 73100 Lecce, Italy

**Keywords:** Biomedical materials, Biomaterials - cells, Drug delivery

## Abstract

Ubiquitous in nature, polyamines (PAs) are a class of low-molecular aliphatic amines critically involved in cell growth, survival and differentiation. The polycation behavior is validated as a successful strategy in delivery systems to enhance oligonucleotide loading and cellular uptake. In this study, the chemical features and the functional roles of the PA spermidine are synergistically exploited in the synthesis and bioactive functionalization of SiO_2_-based structures. Inspired by biosilicification, the role of spermidine is assessed both as catalyst and template in a biomimetic one-pot synthesis of dense silica-based particles (SPs) and as a competitive agent in an interfacial reassembly strategy, to empty out SPs and generate spermidine-decorated hollow silica nanoporous pods (spd-SNPs). Spermidine bioactivity is then employed for targeting tumor cell over-expressed polyamine transport system (PTS) and for effective delivery of functional miRNA into melanoma cells. Spermidine decoration promotes spd-SNP cell internalization mediated by PTS and along with hollow structure enhances oligonucleotide loading. Accordingly, the functional delivery of the tumor suppressor miR-34a 3p resulted in intracellular accumulation of histone-complexed DNA fragments associated with apoptosis. Overall, the results highlight the potential of spd-SNP as a multi-agent anticancer therapy.

## Introduction

SiO_2_-based materials have drawn great attention for biomedical application^1–3^ due to their attractive features of low toxicity, biodegradability and biocompatibility^4–8^ and versatile properties, such as large surface area, long-range ordered porous structure, high mechanical stability and tunable surface chemistry^9,10^. Traditional chemical methods for preparing SiO_2_-based materials have resulted in great success; however, these chemical synthesis approaches typically require harsh conditions, such as high or low pH, high temperatures and/or pressures, use of toxic and/or expensive organic solvents, as well as multiple steps and complex protocols^11,12^. In nature, biosilicification is a sophisticated process by which living organisms produce, at ambient conditions, SiO_2_-based hierarchical structures and multiple morphologies with precise nanoscale control^13–15^. Essentially, there are two main steps by which organisms control the self-assembled hierarchical organic/inorganic structures: first the organic matrix acts as template, and afterwards the inorganic material nucleates, grows up and settles, arranging the biostructure. The high degree of integration among the organic/inorganic sub-units preserves the intrinsic characteristics of the single component, displaying additional properties and advantages, due to the synergistic effect between the different structural entities^16^. Inspired by nature, SiO_2_ particle formation has been designed for diverse co-precipitating and nucleating biological and/or biomimetic agents; at last, these amine-bearing molecules provide, under mild and environmentally friendly conditions, a successful template for the synthesis of SiO_2_-based hybrids^17,18^.

In vitro, different polyamines, such as co-polypeptides with high block ratio of Lys and Phe, polyallylamine, polyethylenimine, poly(acrylamide-co-2-(dimethylamino) ethyl methacrylate or amine terminated dendrimers have been proposed as templates for SiO_2_ formation^19–24^.

Systematic model studies on silicification have been also performed evaluating the role of the naturally occurring short-chain polyamines putrescine, spermidine and spermine^25,26^.

The role of amine species on the condensation of silicic acid systems and the characterization of the resulting product yield have been detailed discussed and demonstrated^27^, nonetheless the integration of their chemical and biological features in composite materials for nanomedicine application is still poorly investigated.

Widely present in living organisms, polyamines (PAs) are a group of aliphatic amines essential for the regulation of cell proliferation and differentiation^28^. PA levels and composition vary between species, the most common of which are putrescine, spermidine and spermine, followed by cadaverine and 1,3-diaminopropane^29,30^. The intracellular demand, highly regulated by metabolic pathways, is maintained through the combination of PA uptake from the extracellular matrix, endogenous biosynthesis, catabolism and excretion^31,32^. PA metabolism dysregulation is involved in many disease conditions; an increased PA concentration^32^ and an overexpression of the polyamine transport system (PTS), a specific energy-dependent and saturable transport system for the transport of exogenous PAs, are often found in various cancer types^33–35^. Consequently, PA pathways have been considered as a potential target for chemotherapeutic agents. Under physiological pH and ionic strength conditions, protonated PAs represent a target of interaction of negatively charged macromolecules, such as DNA, RNA, ATP, phospholipids and certain proteins^36^. The ability to bind nucleic acids and proteins has long been shown to be involved in modulation of gene expression and enzyme activities, in activation of DNA synthesis and transcriptional processes, in the stabilization and remodeling of the chromatin structure and in DNA protection against external agents^36,37^. The high affinity of PA towards oligonucleotide has been a promising approach for oligonucleotide-based therapeutic strategy, since it enhances oligonucleotide biological and biophysical properties, addressing some issues such as poor cell penetration, degradation and low affinity for their targets^38,39^.

In cancer, NPs loading polycationic charges represent a valid strategy for oligonucleotide-based drugs or diagnostics. NPs per se may determine cancer targeting and accumulation since they undergo the “enhanced permeability and retention (EPR) effect” in cancer tissues, where the fenestration of small blood vessel allows permeability^40^. The EPR effect is the rationale for marketed NP-based formulation of old drugs, such as Abraxane, a protein-stabilized nanoparticle formulation of paclitaxel and many others^41^. Cationic PAs loaded on NPs may enhance the cancer targeting properties for their aforementioned features, particularly when the cargo is represented by nucleic acids.

Among oligonucleotides, RNA interfering agents, such as miRNAs and siRNAs, are important biopharmaceuticals and their pharmacological/toxicological activity may be deeply influenced by the delivery tool. MiRNA-34a (miR-34a), downregulates several oncogenes and is lost or down-expressed in many cancers^42^. MRX34, a miR-34a mimic in a liposomal NP formulation, reached the phase I clinical trial against solid tumors. Unfortunately, the trial was halted for the occcurring of severe adverse events, which could potentially be attributed to the liposome carrier^43^.

In this study, the multifunctional roles of PAs are synergistically employed in the design of a bio-functional delivery platform system.

The chemical features and functional roles of the PA spermidine (spd) are specifically applied in the synthesis, functionalization and bioapplication of SiO_2_-based particles. The spd is indeed conceived as the catalyst and template for SiO_2_ precipitation in a biomimetic one-pot synthesis of dense organic/inorganic silica particles (SPs).The spd ability to precipitate composite nanostructured SPs is here investigated by varying synthesis parameters and characterized by TEM, AFM, SEM, DLS and FT-IR. Particle size homogeneity is optimized by tuning precipitation conditions such as temperature, time, organic and inorganic reagent concentration and ratio.

Hollow structures are produced from dense SPs via a smart reassembly route. Free spermidine added in a dispersion of SPs guides a competitive interfacial reassembly, decorating particle surface and emptying out particles. Hollow spermidine-decorated silica nanoporous pods (spd-SNPs) are thus obtained, avoiding the use of the common hard- and soft-core templating methods, such as the formation of a shell over the sacrificial scaffold subsequently removed by calcinations or solvent etching of the organic components^44–46^.

The theoretical basis of the interfacial reassembly method to synthesize hollow particles involves the reunderstanding of hybrid organic/inorganic SiO_2_ structure at the nanoscale level. Herein, the synthesis of both dense and hollow particles is governed by the same molecule. In brief, spermidine acts as both templating and competitive agent in a two-step reversible –assembly-and-reassembly strategy. It is already known that hybrid SiO_2_-based nanocages with a hollow cubic or spherical core, mesoporous shell with organic moiety incorporated frameworks and tunable surface, show enhanced drug loading capacity and responsive drug release, and are able to significantly increase therapeutic outcomes while reducing side effects^47–49^. Accordingly, SPs and spd-SNPs are tested for their bioactivity. Uptake efficiency, cytotoxicity, oxidative stress (OS) and the ability to deliver a functional miRNA are here investigated to assess the role of spermidine conjugation as an alternative delivery approach, so as to efficiently penetrate into tumor cells and release functional oligonucleotides. The oncosuppressor miR-34a-3p loaded in spd-SNP is delivered in MeWo cells resulting in intracellular accumulation of histone-complexed DNA fragments associated with apoptosis. Since spermidine holds extraordinary binding affinity and specificity towards both oligonucleotide and PTS, we expect that hollow spermidine-decorated SNPs could offer a novel solution as a promising oligonucleotide delivery bioactive platform for anti-tumor therapies.

## Results and discussion

**Figure 1:**
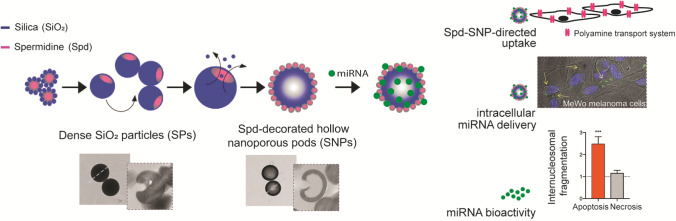
Study key investigations. Spermidine (Spd): role of catalyst and template in a one-pot synthesis of dense silica particles (SP); —spd-guided interfacial reassembly strategy: generation of hollow spd-decorated silica nanoporous pods (SNP); —polyamine transport system involvement in cellular uptake of spd-SNP; —spd-SNP: functional miRNA delivery system in melanoma cells, successful proof of concept.

Organic precursors represent the template capable to control the composition, the shape, the organization and, ultimately, the properties of the inorganic component precipitation in biomineralization process^50,51^. Specifically, these molecules have three main functions: (i) confinement to control particle size, (ii) activation to govern mineral sources concentrations and (iii) templating to guide particle morphology^52^. Investigating the interaction mechanism between the inorganic component and the biomolecules is essential to provide a predictive framework for a fine-tunable synthesis of novel nanostructured materials as well for a targeted post-synthesis structure modification and functionalization^26,53^. The multifunctional role of the organic precursor spermidine in the design and in the bioactivity of oligonucleotide delivery system is here studied following a combined approach (Fig. 1).

### One-pot synthesis of dense SPs

The nucleophilic hydroxyl or deprotonated silanol groups attack in alkaline medium the silicon atoms in alkoxysilane and/or neutral silanol, in hydrolysis and condensation reactions, respectively. Steric and inductive factors affect the polymerization rate, but, in general, the polymerization of silanes is very slow if no catalysts are used. Amine base catalysts are typically used to enhance the occurrence of condensation reactions creating more ramified structure^54^. Alcoxide condensation is directly influenced by the employed amine catalyst, thus the nanostructure of the resultant product reflects the kinetics of the amine-guided condensation reactions.

SiO_2_ particles are typically formed in two steps: small primary particles (< 6 nm) are shaped through nucleation, growth and ripening, and then the final particles grow through aggregation. The reaction medium is usually homogenous in the early stages of the polymerization, and starts to be heterogeneous as either the condensation or the aggregation proceeds. This leads to phase separation and produces a colloidal suspension of particles in a liquid (sol). Particles with sizes less than 20 nm are not colloidally stable^54,55^ within the polymerizing solution and therefore they tend to aggregate before completion of the ripening process.

Spermidine ability to act both as catalyst and as template in SiO_2_ precipitation and growth has been here investigated and characterized via TEM and AFM imaging. In aqueous solution, spermidine aggregates (1.6 mM)^56^ initiate the condensation of tetraethyl orthosilicate (TEOS) (20 mM), controlling small primary hybrid particle growth and/or ripening; the structural growth and the morphological features are strictly directed by spermidine chemical behavior and concentration.

During the early hydrolysis stage, the silicate ions tend to adsorb onto the organic template structure^57^. The silanol groups of inorganic component are attracted through ionic interaction towards organic amino groups, which finally act as catalyst and facilitate the condensation reaction^58^. Particle formation could follow either a diffusion or deposition model according to the specific reaction condition. This suggests that silicic component (oligomers or 2–3 nm particles) diffuses throughout the inner template, whereas larger particles (~ 10 nm) diffuse only through the outer template corona, before depositing themselves to form thick-shelled hollow spheres^59^.

**Figure 2:**
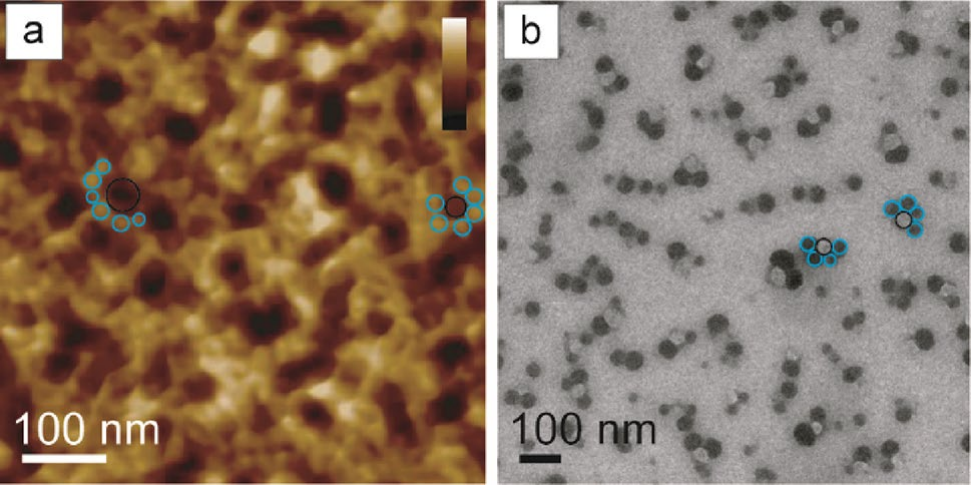
Spd-guided one-pot synthesis of dense SPs. At time 0, primary SiO_2_-based nuclei arrange themselves designing a flower-like organic/inorganic pattern, highlighted by black and light blue circles in **(a)** AFM image (height channel, data scale 0–4 nm, from black to white) and **(b)** TEM image.

In the proposed spermidine-guided one-pot synthesis of dense SiO_2_ particles (SPs), the primary SiO_2_-based nuclei arrange themselves designing a flower-like pattern, immediately after mixing organic and inorganic reagent (time 0) (Fig. 2).

**Figure 3:**
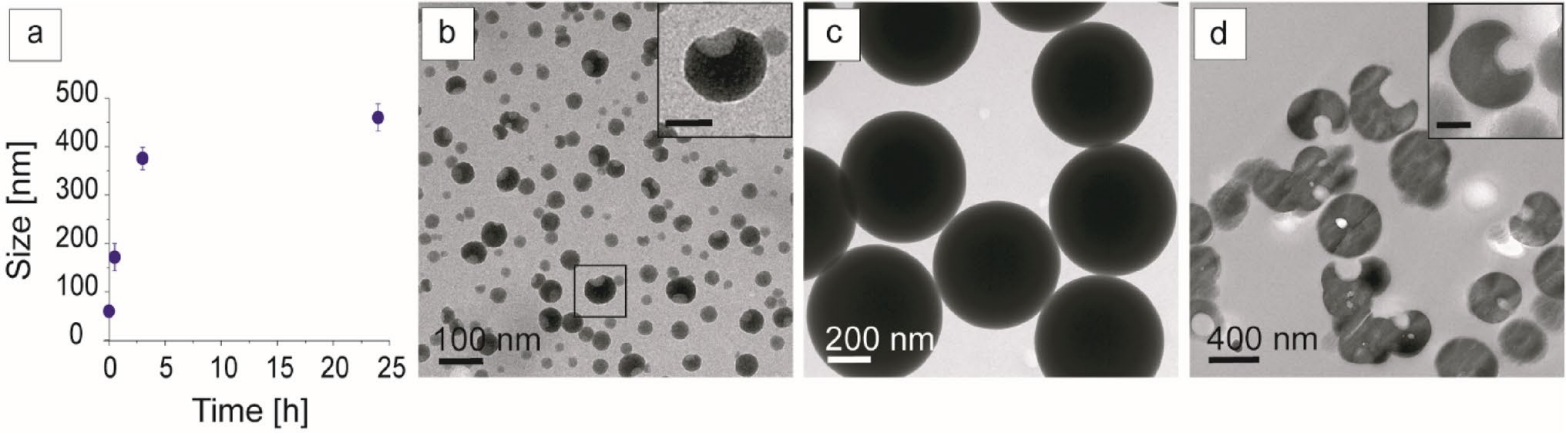
Time-dependent size growth of SPs (reaction conditions: T, 20 °C; spd, 1.6 mM; TEOS, 20 mM). **(a)** Plot of NP size growth. Mean values and SD data are from measurements of about 50–100 particles for each sample. TEM images illustrate SP growth dynamic: (**b, b** inset) after 5 min hybrid nanostructures present a *Pac-Man*-like morphology (inset scale bar 50 nm); **(c)** SiO_2_ deposition proceeds forming, after 3 h, SPs of 377 ± 23 nm. (**d, d** inset) TEM images of SP ultramicrotome sections at 24 h show the conserved inner structural *Pac-Man-*like pattern (**d**, inset scale bar 200 nm).

The intermolecular interactions shorten the distance between growing SiO_2_ nuclei, facilitating their aggregation and giving rise to a growth dynamic (Fig. 3) that rapidly proceeds to the formation of nanostructures presenting a *Pac-Man*-like morphology (60.0 ± 10.4 nm, average diameter) (Fig. 3a,b). The spontaneous process continues following this pattern of aggregation and growth. At 3 h, SPs are characterized by a homogeneous size distribution (377 ± 23 nm, average diameter in Fig. 3a,c) with the constitutive *Pac-Man-*like morphology preserved. At 24 h, SiO_2_ deposition continues further increasing SP size. The 70-nm thick SP ultra-microtome sections (Fig. 3d) confirm the inner structural conformation.

### Effect of experimental conditions on SP size

The concentration of reagents, the reaction time or the temperature are varied one by one to optimize synthesis protocol (Figure S1), allowing SP diameter control from 180 to 800 nm. Particle size growth, always associated to larger polydispersity values and denser structures, is achieved by increasing the TEOS concentration, the time or temperature of reaction. The concentration of TEOS, as inorganic source of SiO_2_-based primary nuclei, influences directly proportional particle size and final yield^60^. Over time, this SiO_2_-based primary nuclei stop growing and start to coalesce, similarly increasing particle size^61^. Temperature affects the increment of the particle size by promoting hydrolysis and polycondensation reactions^62,63^. In order to address particle size decrease, spermidine concentration is reduced as it represents the amine-bearing component playing the role of catalyst and template. A restriction in spermidine amine functional group leads to particles with smaller size and irregular morphology^61^. Well-dispersed dense SPs with a homogenous size distribution (mean diameter, 377 ± 23 nm) are obtained after 3 h of incubation of 20 mM TEOS and 1.6 mM spermidine at 20 °C.

### Preparation of hollow spd-SNPs via interfacial reassembly

The synthesis approaches of hollow mesoporous hybrid organosilicas mainly include hard-core templating methods, liquid-interface assembly and interfacial reassembly and transformation methods. Versatile organic–inorganic hybrid frameworks with high homogeneity and well-controlled structure can be obtained by modulating the synthesis conditions.

**Figure 4:**
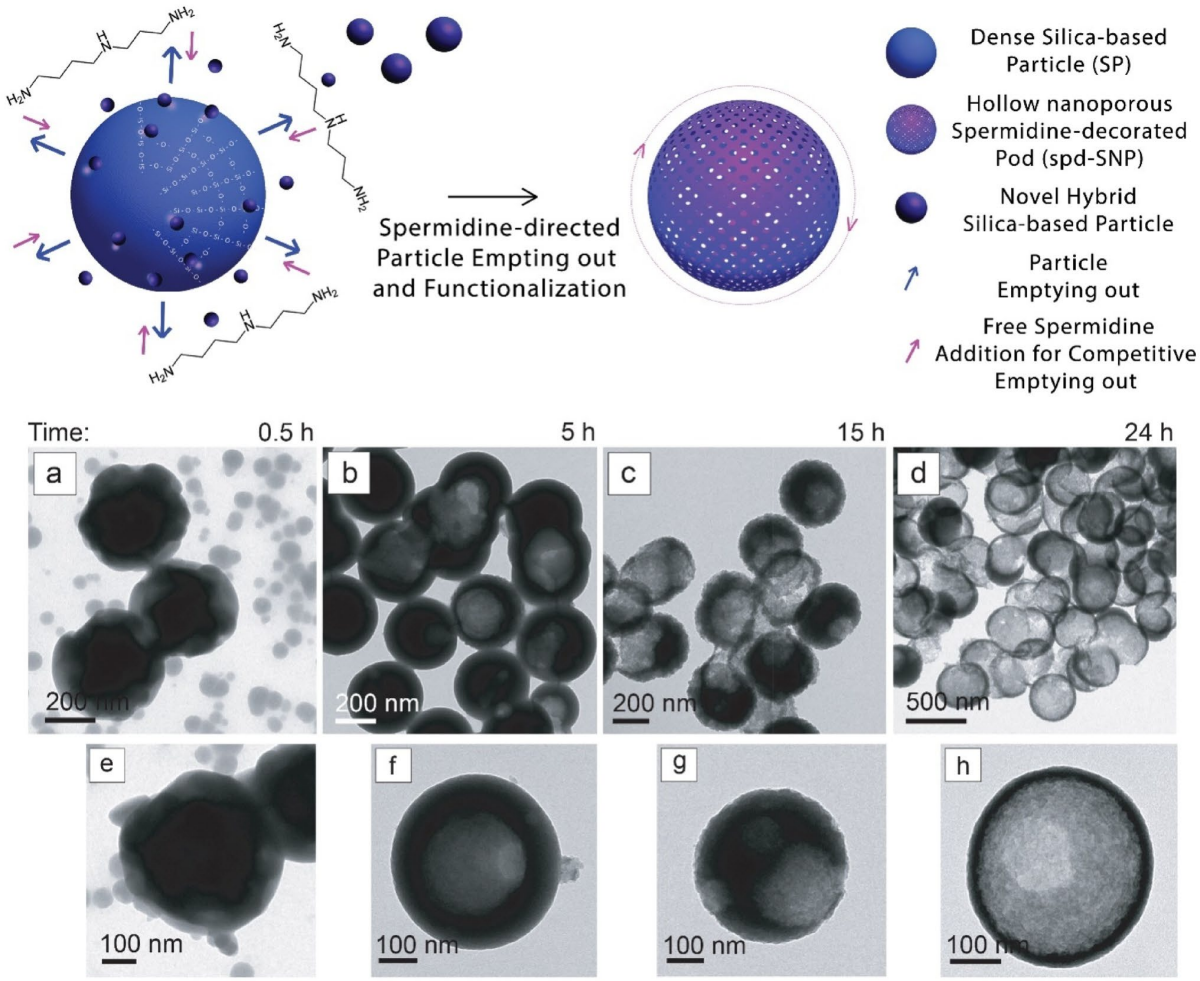
Upper panel: sketch of spd-SNP synthesis: dense SPs are here synthesized to prepare hollow spd-SNP via interfacial reassembly strategy promoted and guided by the competitive action of free spermidine in solution. Lower Panel: time-dependent hollow spd-SNPs formation from dense SPs: TEM images of hollow spd-SNPs after 30 min (**a, e**), 5 h (**b, f**), 15 h (**c, g**) and 24 h (**d, h**) stirring.

To overcome the need of pre-synthesized hard cores or unstable droplets, an attractive smart approach is represented by the conversion of dense to hollow particle via interface-reassembly and transformation^64^. Accordingly, dense SPs (mean diameter of 377 ± 23 nm) are here used to prepare hollow SNP via a smart interfacial reassembly promoted and guided by the competitive action of free spermidine in solution. The electrostatic interaction between spermidine protonated amino groups and negatively charged SP surface induces silica dissolution and guides the mechanism of structural reassembly of the inner hybrid organic/inorganic component into novel hybrid structures (Fig. 4, graphical sketch).

The volume of the hollow cavities and the shell thicknesses is tuned by varying dissolution and reassembly time. To understand the dynamic of hollow SNP formation via interfacial reassembly strategy, the SP incubation step with free spermidine is investigated at different time points (Fig. 4a–h).

A number of additives such as dendrimers and polyelectrolytes have been already shown to enhance silica dissolution. Similarly, supplementary free spermidine in aqueous solution promotes SP inner component dissolution via amine chemical attraction and interaction. Dissolved material reassembles itself in the form of novel hybrid organic/inorganic *Pac-Man*-like structures with a mean size diameter of 60 ± 15 nm (Figure S2).

In details, after 30 min stirring, particle dissolution is confirmed by the presence of novel organic/inorganic aggregates and by a modification of SP morphology and structure, which exhibits a super-assembled surface with an increased whole size dimension from 377 ± 23 nm to 450 ± 35 nm (Fig. 4a,e). Dissolution proceeds over time, leading after 15 h to hollow spherical structure with nanoporous surface (spd-SNPs) and a mean size dimension of 315 ± 20 nm (Fig. 4c,g). After 24 h (Fig. 4d,h), hollow silica particles clearly present a reduced shell thickness.

**Figure 5:**
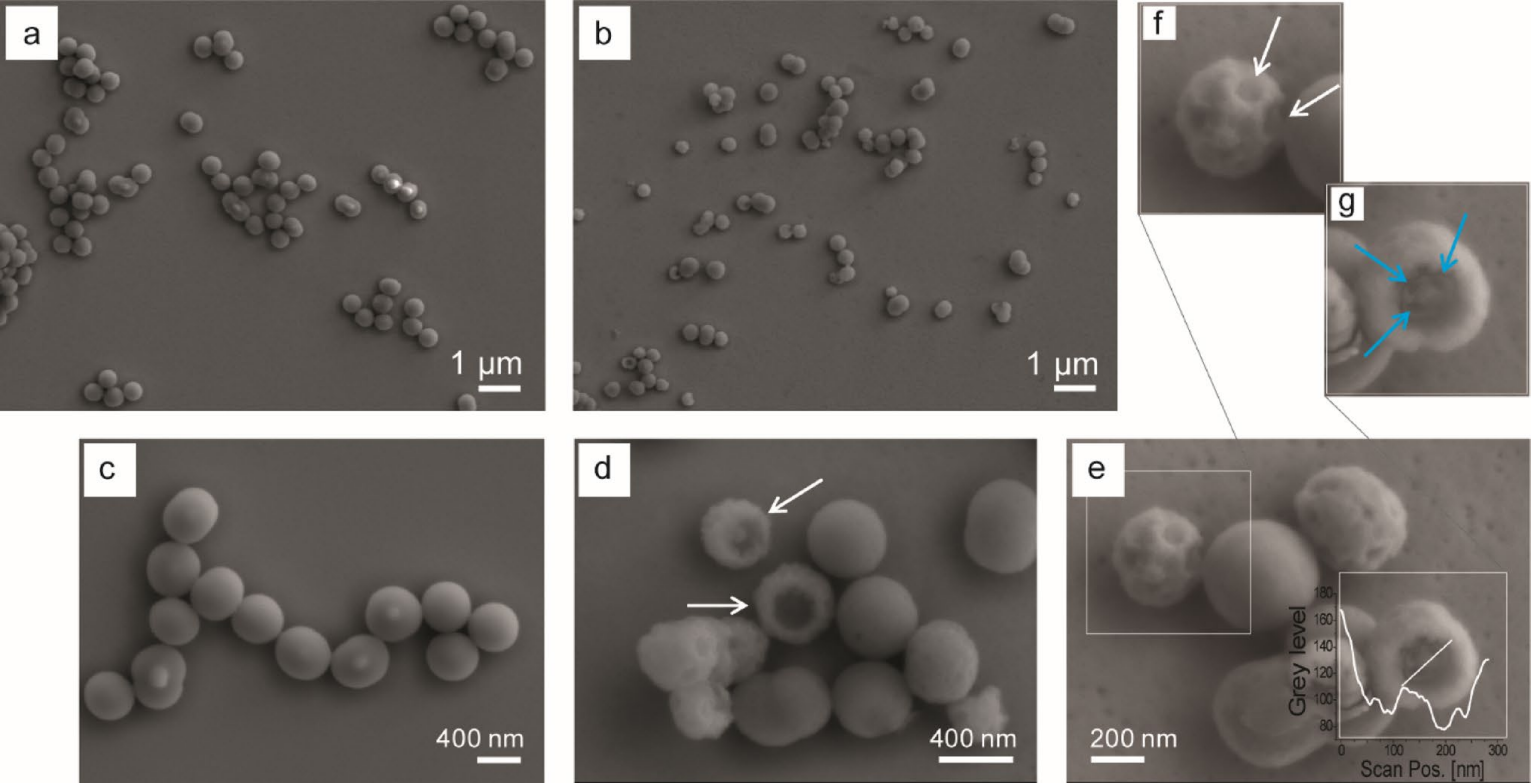
Low magnification SEM images in **(a)** and **(b)** show regular spherical morphology of both SPs and spd-SNPs. Higher magnification images evidence SP smooth surface **(c)** and hollow spd-SNP nanoporous surface (white arrows in **d,f**). In **(e)**, the representative cross section profile (corresponding to the white trace position) describes a peculiar pit (width 190 nm) morphology, revealing the inner pattern with four lobes (width 20–30 nm, indicated by light blue arrows **(g)**) surrounding a central stigma (width 60 nm).

Both SP and spd-SNP are homogeneously dispersed (Fig. 5a,b). In Fig. 5c, SEM imaging reveals the dense structure and the smooth surface of SP. Conversely, the hollow structure and the nanoporous surface of spd-SNP is well exemplified in Fig. 5d,e (and relative magnification Fig. 5f,g).

The reassembly strategy based on a competitive precipitating agent action, enables a controllable tuning of voids and pore size, allowing a targeted cargo of molecules with specific molecular weights^65^.

To confirm the competitive role of spd in the reassembly route, the structural evolution of SPs is monitored in absence of additional spermidine. After 15 h, only a small amount of SP inner structure tends to dissolve, leading to a lower SP emptying (Figure S3). Actually, the dissolved component reorganizes and reassembles onto the outer SP shell, lastly increasing the particle size from 366 ± 23 nm to 530 ± 26 nm, as assessed by EM. Besides, ICP-OES elemental analysis is performed to quantify SP inorganic component removal after SP incubation in extra 3.2 mM spermidine aqueous solution or in UPW (S8). Data show a lower presence of silicon in spermidine-treated particles (pellet:supernatant ratio, 0.95) if compared to those stirred in water only (pellet:supernatant ratio, 2.30).

### Spermidine decoration of hollow SNP

**Figure 6:**
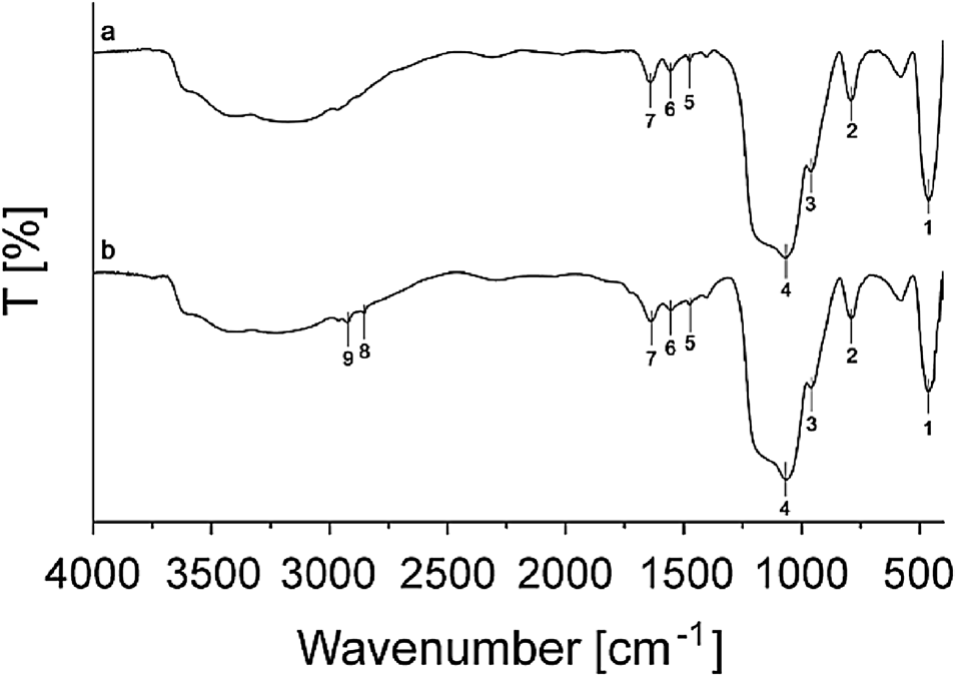
FT-IR characterization of **(a)** SPs and **(b) **spd-SNPs. The peak labels correspond to the following wavenumber: 1 = 468 cm^−1^, 2 = 790 cm^−1^, 3 = 960 cm^−1^, 4 = 1066 cm^−1^, 5 = 1474 cm^−1^, 6 = 1560 cm^−1^, 7 = 1638 cm^−1^, 8 = 2852 cm^−1^, 9 = 2923 cm^−1^.

Spd-SNPs are further characterized by FT-IR analysis and the amount of spermidine is quantified with ninhydrin assay. The IR spectra of SPs and spd-SNPs are reported in Fig. 6. The broad peak from 3500 to 3000 cm^−1^ is attributed to O–H stretching and to the retained water molecules, as well as the peak at 1638 cm^−1^ to the bending of O–H. The intense peaks at 1066 cm^−1^, 790 cm^−1^ and at 468 cm^−1^ are attributed to the stretching of Si–O-Si bond, whereas the peak at 960 cm^−1^ is assigned to Si–OH bond stretching. Concerning the presence of spermidine in the matrix of SiO_2_, and/or onto the surface of the particles, some peaks confirmed the presence of the functional groups. In particular, the peak at 1560 cm^−1^ is assigned to vibrations of N–H bond of amino groups and the peaks at 2923 cm^−1^ and 1474 cm^−1^ are attributed to stretching and bending vibrations of aminopropyl group. All peaks are identified in two preparations; anyway, the spd-SNPs (b) exhibited more pronounced peaks at 2852 cm^−1^ and 2923 cm^−1^ for symmetrical and asymmetrical stretch of CH_2_, confirming the outer layer of grafted PAs.

Additionally, ninhydrin colorimetric assay, often used to detect residual free amines in biochemical synthesis^66^, is here run to quantify spermidine concentration in SPs and spd-SNPs. The amount of amine present in 100 µg SPs and spd-SNPs are respectively 0.1 mM and 0.2 mM. Functionalization step leads to extra spermidine integration, which finally consists in 29% (w/w) of the particle mass.

## Functional studies

### Cellular uptake

**Figure 7:**
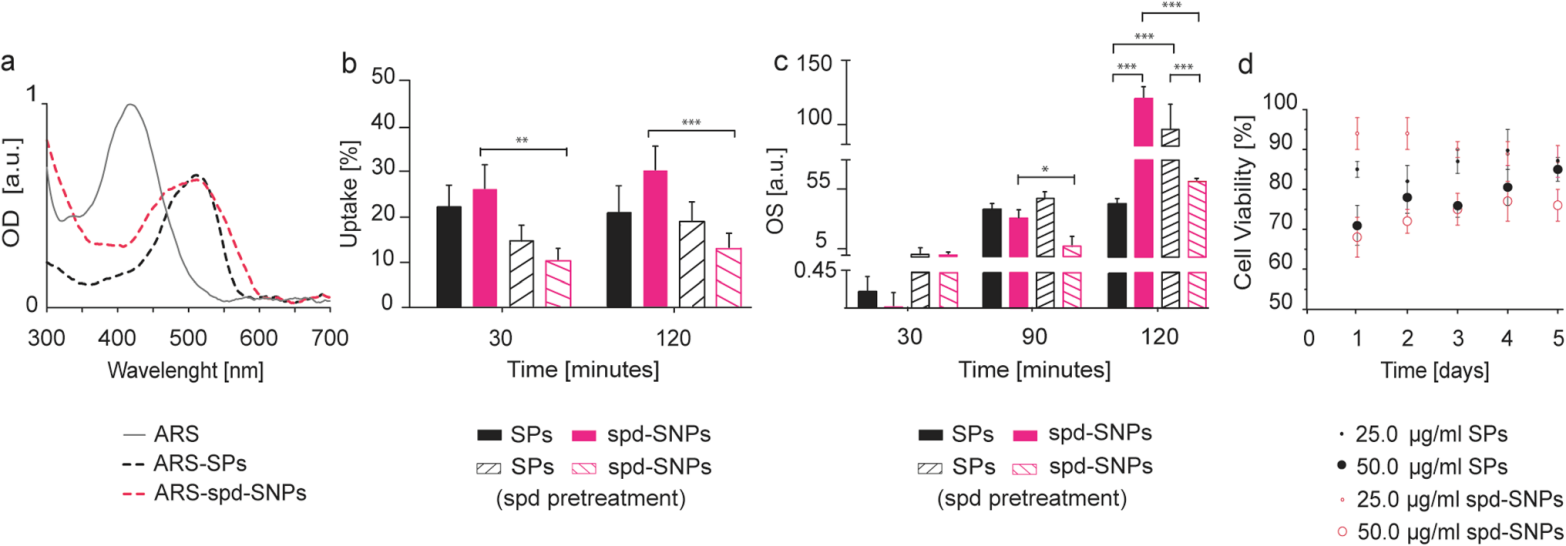
**(a)** Absorption spectra of ARS, ARS-SPs, ARS-spd-SNPs performed in water show particle ARS-labelling. **(b)** SPs and spd-SNPs cellular uptake quantified after 30 min or 2 h of 50 μg ml^−1^ particle exposure, w/o spd pre-treatment. **(c)** OS levels after 30, 90 min or 2 h of 50 μg ml^−1^ particle exposure, w/ and w/o spd pre-treatment. **(d)** Time- and dose-dependent cell viability over 4 days after 24 h incubation with 25 or 50 μg ml^−1^ SPs (blue) or spd-SNPs (pink). Two-way ANOVA followed by Bonferroni post hoc test was used for statistical significance (*** p < 0.001, ** p < 0.01, * p < 0.05). For all the analysis, data are presented as mean values ± SD of three experiments (n = 3).

SPs and spd-SNPs have been labeled with Alizarin Red-S (ARS), an anthraquinone dye, to track and study particle internalization (Fig. 7). Recently, SiO_2_ nanoparticles decorated with amino groups have been functionalized with alizarin complexone via amine bond and, the compatibility for biomedical application has been assessed^67^. An ARS staining of both SPs and spd-SNPs has been successfully performed. Particle ζ potential, at pH 6.5, turns into more negative values, from − 21.6 ± 4.4 mV to − 31.7 ± 4.5 mV for SPs and from − 15.5 ± 4.6 mV to − 27.3 ± 4.6 mV for spd-SNPs, due to the negatively charged functional groups of ARS. It is known that ARS can exist in three different forms depending on solution pH value. In neutral form, ARS absorbs at 433 nm, in monoionized form at 526 nm and, in dionized form at 567 nm^68^. ARS shows a yellow color in pH < 5 solutions and a violet color at pH of ~ 11^69^. ARS 0.02% (w/v) in UPW/0.015% ethanol exhibits a maximum absorption peak at 445 nm. ARS labeled particles, suspended in UPW, turns from a violet to purple-red color and exhibits a bathochromic shift to 530 nm (Fig. 7a), supporting that ARS-labelling has occurred and revealing its monoionized form onto particle structure. Quantitative evaluation of cellular uptake (Fig. 7b) reveals a comparable uptake efficiency for both SPs and spd-SNPs after 30 min (22.1 ± 5.1)% and (25.6 ± 5.6)%, respectively, which slightly increases after 2 h exposure in particular for spd-SNPs ((20.8 ± 6.3)% for SPs and (30.0 ± 5.6)% for spd-SNPs)).

To assess the involvement of PTS in particle internalization, cell pre-treatment with free spermidine is performed, aiming at blocking the transport system. Specifically, if PTS selectively takes part to particle uptake, it is expected that, after blocking its function, cellular uptake would quantitatively decrease. Data confirm PTS involvement in particle internalization, mainly for spd-SNPs, indicating an active uptake via spermidine-PTS affinity^70,71^.

After spermidine pre-treatment, the internalization of spd-SNPs is considerably influenced resulting in a 60–65% fall (p < 0.001). Uptake data show a decreasing trend from (25.6 ± 5.6)% to (9.3 ± 2.9)% (p < 0.01), after 30 min exposure, and from (30.0 ± 5.6)% to (12.6 ± 3.3)% (p < 0.001), after 2 h exposure. The decreasing trend in cellular uptake is not observed for SPs, since they do not hold the additional spermidine moiety (from (20.8 ± 6.3)% to (18.8 ± 4.4)% after 2 h of exposure), further validating the role of spermidine functionality in PTS-mediated particle uptake.

### Early ROS generation

Although SiO_2_ nanoparticles are generally considered as non-toxic nanomaterials, several in vitro studies showed that amorphous SiO_2_ nanoparticles reduce the viability of several cell types^72–74^ and/or increase ROS production^75–78^; OS is considered the main mechanism in SiO_2_ nanoparticles toxicity^79,80^.

To investigate the involvement of early stress level after 50 μg ml^−1^ NP exposure, ROS generation has been measured 30, 90 min and 2 h after treatment (Fig. 7c). OS is evidenced after 90 min exposure. Fluorescence data processing points out a similar ROS generation of 39.3 ± 4.7 (a.u) and 30.1 ± 8.0 (a.u.) for SPs and spd-SNPs, respectively. Remarkably, a significant difference in particle type-dependent cellular ROS production is observed after 2 h exposure. Incubation with 50 μg ml^−1^ spd-SNPs, compared to SPs, determines a three fold higher ROS production (124.5 ± 11.7 (a.u.) against 44.3 ± 4.5 (a.u.), p < 0.001). The intracellular ROS concentration over 2 h exposure is followed by a cytotoxicity level of (29.6 ± 2.9)% and (22.4 ± 3.8)% (d.n.s.), for SPs and spd-SNPs respectively. Indeed, these results indicate that SPs and spd-SNPs are able to induce OS into MeWo cells, and since ROS is known to induce DNA damage and to interact with cellular membranes, OS is probably enrolled with cell death^81^.

ROS generation and cytotoxicity are also evaluated after spermidine pre-treatment to confirm the role of PTS as a selective way for particle internalization. Spermidine pre-treatment affects both ROS generation and cytotoxicity, particularly referring to spd-SNP exposure. After 2 h spd-SNP incubation with free spermidine pre-treated cells, a significant ROS level fall is observed, if compared with not pre-treated control (from 124.5 ± 11.7 (a.u.) to 62.7 ± 2.7 (a.u.), p < 0.001), pointing out an uptake efficiency decrement. The OS reduction is followed by a decrease of cytotoxicity from (22.4 ± 3.8)% to (7 ± 2.9)%, confirming the role of PTS in spd-SNPs internalization. These experiments suggest that spermidine functionalization selectively acts on particle transport into MeWo cells by a PTS-mediated endocytosis process, resulting fully consistent with data reported by recent studies^70,71^.

### Cytotoxicity

SiO_2_ particles induced time-, dose- and cell-dependent cytotoxicity^82,83^. Cytotoxicity effects are generally observed, for amorphous SiO_2_ particles, only at or above the concentration of 25 μg ml^−1^^84^.

The dose-dependent cytotoxicity of SPs and spd-SNPs is here assessed after 24 h exposure in standard cell culture conditions (Fig. 7d). Compared to the untreated control (100% cell viability), 6.25 and 12.5 µg ml^−1^ of both SPs and spd-SNPs do not induce any relevant effect. Cell viability starts to slightly decrease only after 25 μg ml^−1^ particle exposure (respectively, to 86.3% for SPs and 93.2% for spd-SNPs) with no great differences observed within 5 days (87.0% and 87.4%) or between particle types. A cytotoxic response is observed at a concentration of 50 μg ml^−1^. After 24 h exposure, cell viability decreases almost similarly, from 100% to (71.4 ± 2.9)% for SPs and to (68.2 ± 3.8)% for spd-SNP treatment.

Five days after the treatment, the viability of cells treated with SPs up-turns from (71.4 ± 2.9)% to (85.0 ± 3.4)%, while under spd-SNP treatment the dose-dependent cell-viability remains almost similar and it varies from (68.2 ± 3.8)% to (75.7 ± 3.8)%. In particular, the viability retrieval of MeWo cells treated with spd-SNPs results 50% lower than the one undergoing SP treatment. Spd-SNP structural and physiochemical properties influence particle toxicity, increasing carrier functional activity in antitumor drug delivery approaches.

### Oligonucleotide conjugation

Nucleic acid adsorption to silica material is attributed to multiple and sometimes diverse driving forces, dealing with electrostatic interaction, free electrolytes in solution and hydrogen bonding between unwound nucleotides and SiO_2_ surface^85–87^. To enhance conjugation efficiency, SiO_2_ materials are typically modified with positively charged organic adjuncts, involving surface labelling with amine groups or coating with metal cations or cationic polymers^88^. PAs are able to bind by electrostatic linkages to many cellular macromolecules, including DNA, RNA, and proteins^36^. Among these, well-characterized efficient transfecting agents are represented by branched or linear polyethilenimine^89^. Herein, hollow nanoporous structure and spermidine molecules, both exposed onto particle surface and intimately present into hybrid structure, are actively enrolled in nucleic acid conjugation. The conjugation of miR-34a-3p is carried out to test the material as a delivery system. For the functional studies, the loading process has been tuned in order to conjugate 100 nM miR-34a-3p in 50 μg/ml spd-SNPs. The conjugation technique provides for a noncovalent binding of the biomolecule, and considers both the loading in the hollow cavities and the loading mediated by spd-miRNA binding affinity^38^. Spd-SNPs hollow structure and surface functionalization improve oligonucleotide loading efficiency by a 10% factor if compared to dense SPs. A variation of the environment nearest to the fluorophore is observed in the emission spectra (Figure S5). Spd-SNP fluorescence curve presents a slight bathochromic shift^90,91^. Noncovalent conjugation strategies, including oligonucleotide/amine-bearing molecule conjugation, are particularly employed for the advantages of a rapid conjugation kinetic, easy tunability and good stability; conversely, noncovalent conjugation site-selectivity is not easily controllable. Considering the contribute of hollow structure in loading mechanism and taking into account the different order of magnitude of miR-34a-3p and spermidine molar concentration involved, miR-34a-3p would not saturate surface amine component.

The spd-SNPs ability to translocate oligonuclotide molecules across biological membranes to human melanoma cells (MeWo cells) is assessed after 24 h incubation. Confocal microscopy imaging shows a good internalization level (Figure S6) thus confirming the role of spermidine-decorated hollow nanoporous structure as a successful strategy to enhance cargo loading and delivery in cellular body, paving the way to future investigations of cell trafficking and release.

### Particle dissolution rate

**Figure 8:**
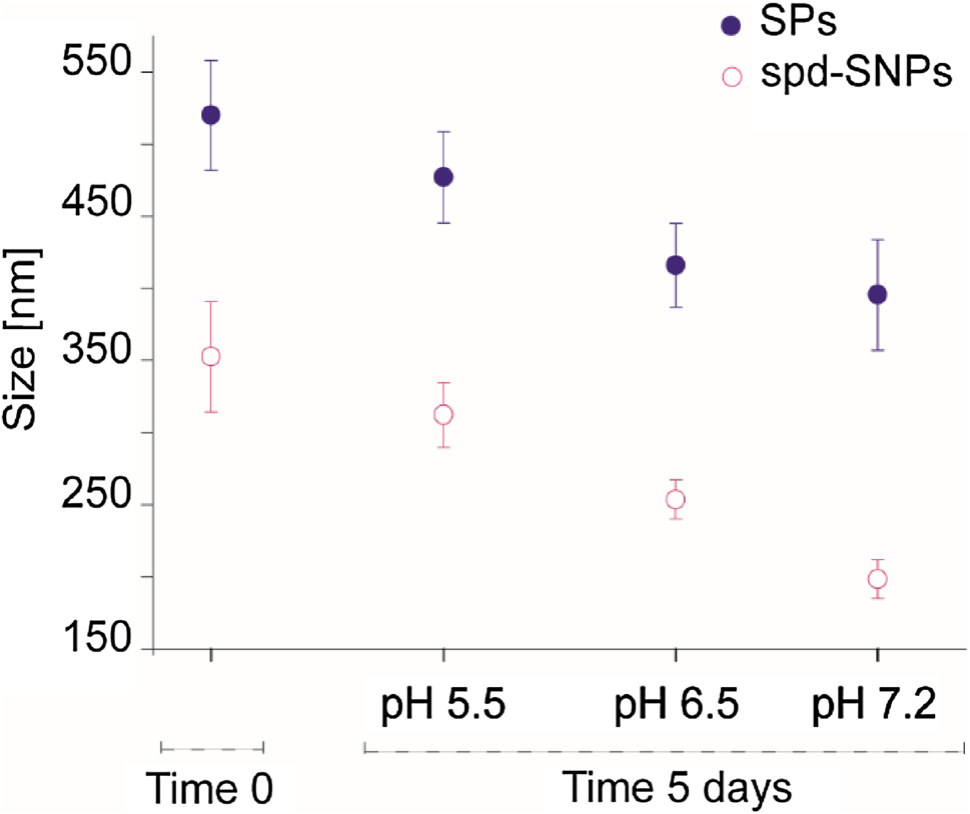
SPs (blue) and spd-SNPs (pink) pH-dependent dissolution. NPs are incubated in PBS at pH 5.5, 6.5 and 7.2 over 5 days. An increase in the polydispersity index (PDI) values due to particle degradation is reported in Table S1.

The dissolution of SiO_2_ nanoparticles is well described in literature and is caused by the hydrolysis of SiO_2_ matrix, which is known to be accelerated at higher pH and temperature^92,93^. Particle dissolution is here evaluated in aqueous environment at pH 5.5, 6.5 and 7.2, typical of the intracellular environment in late endosome, early endosome and cytosol, respectively^94^. DLS analysis is performed to characterize the hydrodynamic diameter after 5 days of incubation at 37 °C (Fig. 8). None aggregation data is recorded, confirming the good stability in aqueous solution. Given that the dissolution rate increases in basic pH conditions^95^, after 5 days the particle size decreases more at pH 7.2, resulting in a diameter reduction of 33.3% for SPs (from 520.0 ± 38.2 nm to 393.5 ± 11.4) and of 43.7% for spd-SNPs (from 352.4 ± 38.4 nm to 198.3 ± 13.4 nm). Surface modification influences the degradation rate. Amino-functionalized mesoporous SiO_2_ particles are well-known to be faster degradated^64^. Moreover, spd-SNP larger surface area, due to the presence of hollow porous structure contributes to a more intense degradation.

### miR-34a-3p delivery and functional activity

**Figure 9:**
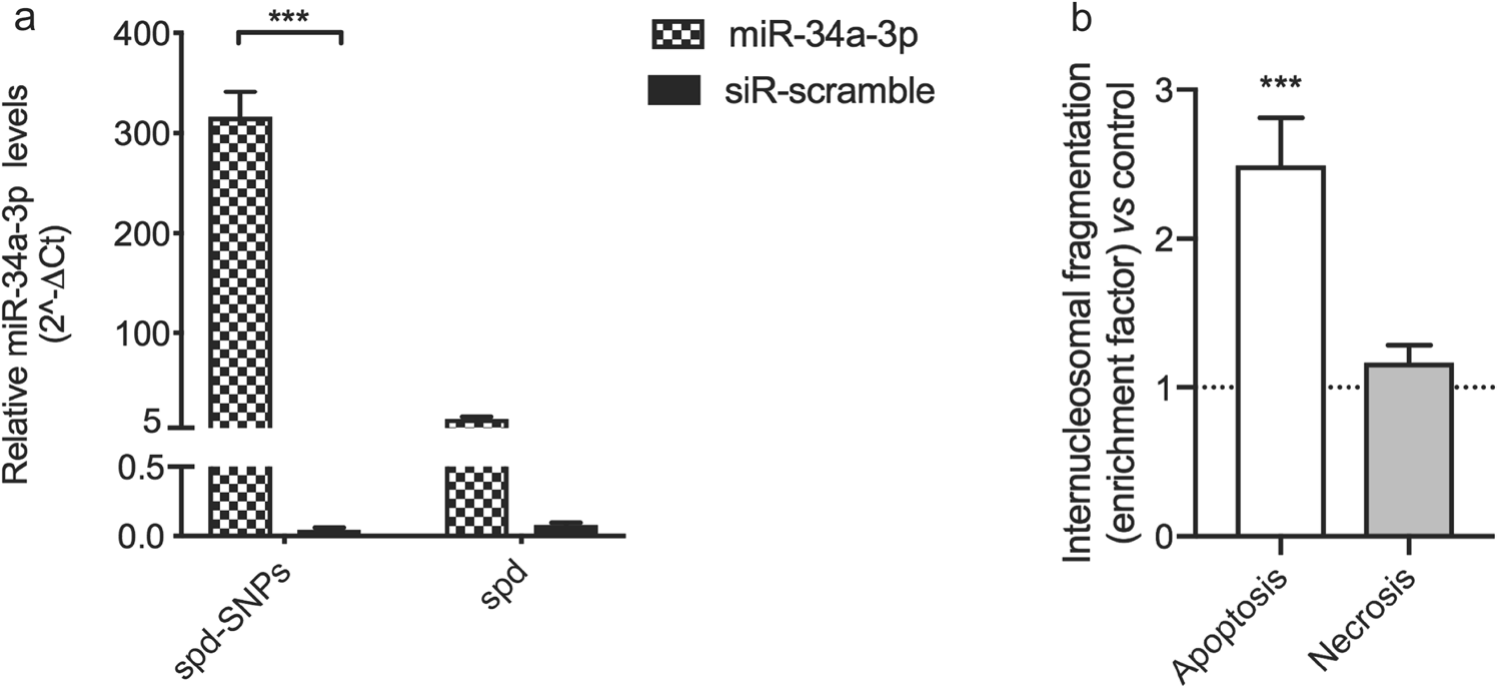
**(a)** Real-time PCR assessment of miR-34a-3p levels in MeWo cell line. Cells were transfected with spd-miR-34a, spd-siR-scramble, spd-SNPs-miR-34a-3p and spd-SNPs-siR-scramble and the intracellular levels of miR-34a-3p were analyzed after 12 h. Values were expressed as mean ± SD from three separate experiments. ***p < 0.001, compared to control (two-way ANOVA followed by Bonferroni’s post hoc test). **(b)** Internucleosomal DNA fragmentation assessed in lysates (apoptosis evaluation) of MeWo cells or in culture medium (necrosis evaluation) after treatment withspd-SNPs-miR-34a-3p or spd-SNPs-siR-scramble (control) for 72 h. Data are expressed as mean ± SD from three separate experiments. ***p < 0.001 as compared to spd-SNPs-siR-Scramble (ordinary one-way ANOVA followed by Bonferroni’s post hoc test).

In Fig. 9, after 12 h of incubation with spd-SNPs loaded with miR-34a-3p, MeWo melanoma cells show a significant increase of miR-34a-3p levels compared to cells treated with siR-scramble-loaded spd-SNPs (316.15 ± 25.09 and 0.44 ± 0.15 fold increase, respectively) (Fig. 9a). A lower and not significant increase in the intracellular levels of miR-34a-3p is instead observed after cell exposure to spd-miR-34a-3p (14.75 ± 1.22 fold increase).

The ability of spd-SNPs to deliver miRNA in a functionally active form, into human melanoma cells, was investigated by evaluating internucleosomal DNA fragmentation, an intracellular marker of apoptosis and extracellular marker of necrosis. MiR-34a-3p is, in fact, recognized as an oncosuppressor and pro-apoptotic miRNA, as reported in different studies^96,97^. Cell exposure to spd-SNPs-miR-34a-3p induced an intracellular accumulation of histone-complexed DNA fragments (apoptosis) by 2.5-fold higher than that obtained from cells transfected with siR-scramble-loaded spd-SNPs. As expected, no significant necrotic death was observed in treated cells (Fig. 9b).

## Conclusions

Bioinspired SiO_2_-based particle synthesis is an appealing alternative to synthetic methods. Recently, top-down approaches have gained considerable attention. From different biomasses, as diatoms microalgae, equisetum and rice husk, different hybrid organic/inorganic SiO_2_-based clusters have been extracted^98–100^. Hence, moved by naturally occurring SiO_2_ based nanostructured biomaterials, biomolecule analogues and/or synthetic amine-bearing molecules have been considered as co-precipitating and nucleating agents in biomimetic synthesis under ambient conditions, in order to provide more tunable materials and scalable manufacturing protocols. Spermidine is the amine-bearing molecule considered to efficiently promote SiO_2_ precipitation in this work. Spermidine chemical properties and functional bioactivity are efficiently integrated in a combinatorial approach. The multiple role of the organic precursor is employed in both the design and bioactivity of the proposed oligonucleotide delivery system.

Spermidine acts both as the organic catalyst and template able to mediate a controllable silicification in aqueous solution and guides an interfacial reassembly process to generate hollow nanoporous silica-based pods (spd-SNPs).

Preliminary functional tests have elucidated a good intake by melanoma cells, showing a lower viability retrieval of melanoma cells treated with spd-SNPs versus those incubated with SPs. Spd-decorated structures have been investigated as a novel class of Trojan horse, to selectively delivery oligonucleotides via the specific energy-dependent and saturable polyamine transport system, overexpressed in cancer cells. Oligonucleotides, including miRNAs, are an innovative category of drugs for the treatment of a vast palette of diseases, but their polyanionic nature limits the cellular uptake. Oligonucleotide cellular transfection and in vivo delivery to target tissues represent two major goals for nanomedicine. As regards cancer therapy, nanoparticle-mediated delivery of therapeutic oligonucleotides may offer several advantages, such as increased cancer tissue selectivity, reduced systemic toxicity and prolonged drug half-life^101–103^.

Due to PAs biological role, spd-SNPs could be further investigated for spermidine pleiotropic effects, which include anti-inflammatory properties, antioxidant functions and enhancement of mitochondrial metabolic function and respiration and for autophagy-dependent mechanisms^104^.

In this context, polyamine-coating has gained technological significance for gene therapy applications^105^, further supporting our spd-SNPs for their activity of nucleic acid precipitation, aggregation and condensation. Moreover, taking advantages of the well-documented polyamine/nucleic acid affinity and silica surface properties, new application scenarios could be opened in solid phase extraction and purification of DNA from complex samples. In conclusion the novel composite pod structure, possibly could lend great potential in serving as toolbox, both in diagnostics and therapeutics, similarly to spheroidal silica cages.

## Experimental

### Materials

Ultrapure water (UPW) was obtained by a Barnstead Smart2Pure Water Purification System (ThermoFisher Scientific, USA). Ethanol, Tetraethyl Orthosilicate 98% (TEOS), Spermidine BioReagent ≥ 98% (spd), l-Glutamine, Dulbecco’s modified Eagle’s medium (DMEM), Fetal Bovine Serum (FBS), Penicillin/Streptomycin Solution, 2’,7’-Dichlorofluorescin diacetate ≥ 97% (DCFH2-DA), Thiazolyl Blue Tetrazolium Bromide 98%, Alizarin Red S (ARS), 4’,6-diamidion-2-phenylindole (DAPI), Phosphate Buffered Saline (PBS) and Paraformaldehyde were obtained from Sigma Aldrich (Germany).

### One-pot synthesis of hybrid SPs

Spermidine aqueous suspension and TEOS dissolved in a 30% ethanol solution were prepared at various millimolar concentrations and separately sonicated at a frequency of 59 kHz for 5 min at 18 °C. Reagent solutions were mixed for 5 s, at 900 rpm, and heated to 40 °C in a microwave system. The mix was then stirred, for 5 s at 900 rpm and incubated for 3, 8 or 24 h at 20 or 25 °C in a temperature controlled water bath. The obtained SPs were precipitated at 10,000 rpm for 10 min and the pellet washed twice with UPW. To produce monodisperse SiO_2_ particles of various controlled size, several experiments, varying the ratio of reactants as well reaction temperature and time, were performed (Figure S1). SPs were then freeze-dried on Alpha 1–2 LD plus CHRIST (Germany) for subsequent processing and functional studies.

### Preparation of spd-SNPs via interfacial reassembly

SPs (400 µg) were resuspended in 1 ml UPW with spermidine (3.2 mM). The mix was stirred for 15 h on Multi Reax vibrating shaker (Heidolph, Germany) at 700 rpm and room temperature, and then precipitated at 13,000 rpm for 30 min on Eppendorf MiniSpin Microcentrifuge (Fischer Scientific, USA). Spd-SNPs were then freeze-dried for subsequent processing and functional studies.

### Characterization of SPs and spd-SNPs

Experimental and instrumental details of methods used for characterization of SPs and spd-SNPs are reported in SI.

### Ninhydrin assay

Ninhydrin assay was performed to determine the concentration of primary and secondary amines in suspension. Ninhydrin reagent was allowed to react with spermidine at concentrations between 0.01 and 3.2 mM. The absorbance at 570 nm was recorded to establish the calibration curve by plotting the optical density versus spermidine concentration.

Particle samples dispersed in water were added to a 2% ninhydrin aqueous solution. The mixture was heated into boiling water for 10 min and after that, it was cooled down to room temperature. The absorbance was measured at 570 nm on a microplate reader. The molarity of unknown concentration of spermidine in 25, 50, 100 and 200 µg of SPs and spd-SNPs was determined by interpolating the measured absorbance to the calibration curve.

### Staining with Alizarin Red-S

A 2% (w/v) ARS in 15% (v/v) ethanol aqueous solution was stirred for 15 min at 800 rpm and then filtered with a 0.2 µm nominal-pore-size cellulose acetate filter. ARS-solution (10 µl)was then added to 500 µg of purified SPs or spd-SNPs, dispersed in 500 µl of UPW, and stirred for 30 min at 400 rpm, at room temperature. Unbound ARS was separated in UPW thrice by centrifugation. NP absorbance spectra were analyzed on microplate reader.

### Cell culture

Human melanoma cells MeWo (ATCC HTB-65) were obtained from the American Type Culture Collection (ATCC). MeWo cells were cultured in DMEM, supplemented with 10% (v/v) FBS, 1% (v/v) l-glutamine, 1% (v/v) penicillin/streptomycin at 37 °C, in a humidified atmosphere containing 5% CO_2_.

### Spermidine pre-treatment

Cell incubation with free spermidine was performed to block PTS and value PTS role in particle internalization. The pre-treatment was performed immediately prior to particle exposure, by adding spermidine (50 µg ml^−1^) and incubating for 30 min.

### Cellular uptake

For particle uptake quantification, MeWo cells were seeded in a 24-well plate at a density of 5 × 10^4^ cells/well and cultured in standard conditions. At 60% of confluence, cells were exposed (for 30 min and 2 h) to 50 μg ml^−1^ ARS-SPs or ARS-spd-SNPs in PBS containing 10% (v/v) of FBS. After the exposure time, the medium was removed, cells were washed thrice with PBS and the absorbance was read at 530 nm in area scan modality. Particle uptake amount was determined by interpolating the recorded optical density values with the calibration curve obtained by SPs or spd-SNPs at known concentration (5, 10, 25, 50, 100 µg ml^−1^).

### Detection of reactive oxygen-species (ROS)

Generation of intracellular ROS was detected by DCFH_2_-DA staining. The non-fluorescent lipophilic DCFH_2_-DA diffuses and crosses the cell membrane. Under the action of intracellular esterases, DCFH_2_-DA deacetylates to form membrane-impermeable DCFH_2_ still non-fluorescent. When DCFH_2_ reacts with intracellular ROS, it gives the fluorescent compound DCF. DCF fluorescence intensity indicates finally intracellular ROS level.

MeWo cells were seeded in 96-well plate at a density of 1 × 10^4^ cells/well; at 60% of confluence, cells were incubated with 25 µM of DCFH_2_-DA at 37 °C, for 30 min and then washed thrice with PBS, to remove not internalized DCFH_2_-DA traces. Photoluminescence, from each well, was measured and recorded as reference of basal OS. Cells were then exposed to 50 μg ml^−1^ SPs or spd-SNPs overtime; ROS levels were monitored every 30 min up to 2 h. DCFH_2_-DA-loaded cells were analyzed in the microplate reader set at 37 °C, with λ_ex_ 504 nm (bandwidth, 9.0 nm) and λ_em_ 529 nm (bandwidth, 9.0 nm). Variation of intracellular ROS level was measured also after spermidine pre-treatment. Further, cell viability was quantified in order to correlate the possible involvement of ROS generation in particle cytotoxicity at 2 h. The data analysis was performed calculating the percentage increase in fluorescence/well by the following formula: [(Ft_30_ − Ft_0_) (Ft_0_ 100)^−1^], where Ft_30_ = fluorescence at time 30 min and Ft_0_ = fluorescence at time 0 min.

### Cell viability

Cytotoxicity evaluation of SPs and spd-SNPs was performed using MTT assay^106^. Cells, seeded in 96-well plate at a density of 1 × 10^4^ cells/well were allowed to reach 60% confluence. 6.25, 12.5, 25 or 50 μg ml^−1^ of SPs or spd-SNPs were dispersed in complete medium, and incubated with cells, for 24 h. After the exposure time, the medium was removed, cells were washed thrice with PBS and incubated with fresh complete medium over the following 4 days for cell viability evaluation at set time points.

200 µl of DMEM with 0.5 mg ml^−1^ of MTT reagent (3-(4,5-dimethylthiazolyl)-2,5-diphenyl-tetrazoliumbromide) was added to each well and was further incubated for 4 h. Blue formazan crystals, formed from MTT by enzymes associated to metabolic activity, were then dissolved with 200 μL of DMSO per well. The plates were gently swirled for 10 min, at room temperature, to dissolve the precipitated salt. The absorbance was measured at 570 nm; wells with complete medium, SPs or spd-SNPs and MTT reagent, without cells, were used as blanks. Cytotoxicity was expressed as the percentage of cell viability, where untreated cells were set to be 100% viable. Given results represent mean values from triplicate measurements.

### Oligonucleotide loading

For the evaluation of oligonucletide loading efficiency, the following scrambled oligonucleotide sequence was used: 5′AAC GTA CTT CGT GTT GAT GAA GAG GA-FAM 3′ (molecular weight, 8074.34 g/mol).

Oligonucleotide (450 nM) was added to a suspension of 200 µg SPs or spd-SNPs. The mix was stirred at 700 rpm for 2 h at room temperature. Oligonucleotide-particles complex was centrifuged at 150,000 g for 30 min on a Beckman Coulter Optima L−100 XP ultracentrifuge (SW 41 Ti Swinging-Bucket Rotor), to remove unbound oligo, to be then quantified. Oligonucleotide loading efficiency was determined by means of NanoDrop-2000 spectrophotometer (Thermo Fisher Scientific, Wilmington, DE, USA). Unbound oligo concentration was calculated by NanoDrop-2000 software, based on the absorbance at 260 nm, on the selected analysis constant and on the baseline correction, and were reported in terms of mass units (ng µl^−1^).

### Transfection of spd-SNPs-miR-34a-3p complex

miScript miR‐34a‐3p mimic was purchased from Qiagen (Hilden, Germany). A negative control siRNA, i.e. siR-scramble (SI03650318, Qiagen, Hilden, Germany), was used in each experiment. Spd-SNPs were initially dissolved in sterile water and then added with miRNA mimic or siR-scramble. Then, the mix was stirred at 700 rpm for 2 h, at RT. 60% confluent cells were incubated for 12 h with spd-SNPs (50 μg/ml) or free spd (0.1 mM) complexed with miR-34a-3p or siR-scramble (100 nM).

### Evaluation of miR-34a-3p levels in MeWo cells

The ability of spd-SNPs to deliver miR-34a-3p was evaluated analyzing intracellular levels of this miRNA by real-time PCR. Purification and extraction of total miRNAs were obtained by using the miRNeasy Mini Kit (Qiagen, Hilden, Germany). The extracted miRNAs were then retro-transcribed by the miScript Reverse Transcription Kit (Qiagen, Germany) and the corresponding cDNA diluted 1:3 in RNase-free water. The miScript SYBR-Green PCR kit (Qiagen, Germany) was used to perform qPCR experiments in triplicate. Signals were detected on the MiniOpticon CFX 48 real-time PCR Detection System (Bio-Rad, Hercules, CA, USA). MiScript Primer Assays specific for hsa-miR-34a-3p (miRBase code: MIMAT0004557) and hsa-SNORD6 were obtained from Qiagen. MiRNA levels were calculated using the Ct method and normalized to the levels of the SNORD6 housekeeping gene, as previously reported^107^.

### MiR 34a-3p-induced apoptosis/necrosis

Apoptosis and necrosis of MeWo cells were assessed after 72 h treatment with spd-SNPs-miR-34a-3p or spd-SNPs-siR-scramble, using the Cell Death Detection ELISA Kit (Ref. 11774452001, Roche, Mannheim, Germany), according to the manufacturer’s protocol. Briefly, treated cells were lysed and centrifuged at 200 × *g* for 10 min. The supernatant of lysed cells, for apoptosis evaluation, and cell culture medium, for necrosis evaluation, were placed into a streptavidin-coated microplate. Then, a mixture of anti-histone-biotin and anti-DNA-POD antibodies was added and incubated at RT for 2 h. After incubation, unbound antibodies were removed from the solution and the nucleosomes were quantified by color development with substrate. Optical density was measured at 405 nm with the Infinite M200 NanoQuant instrument (Tecan, Salzburg, Austria).

### Quantification and statistical analysis

Statistical comparisons of particle uptake e ROS generation were performed using GraphPad Software Prism v5 (San Diego, USA). One way or two-way ANOVA followed by Bonferroni’s multiple comparison test was applied. Data are presented as mean ± SD of technical replica (n = 3); the differences were considered significant for p < 0.05.

## Supplementary information

Supplementary Figures.
