# Supplementary material for: Tailoring of silica-based nanoporous pod by spermidine multi-activity
Source: Sci Rep. 2020 Dec 3;10:21142. doi: 10.1038/s41598-020-77957-4 (PMC7712788; doi:10.1038/s41598-020-77957-4)
Supplement: Supplementary file 1 — Supplementary Figures. [file 41598_2020_77957_MOESM1_ESM.docx]

**Tailoring of Silica-Based Nanoporous Pod by Spermidine Multi-Activity**

Giulia Della Rosa,^1,a^ Riccardo Di Corato,^1,2,b^ Sara Carpi,^3,4^ Beatrice Polini,^3^Antonietta Taurino,^5^ Lorena Tedeschi,^6^ Paola Nieri,^3,4^ Rosaria Rinaldi,^1,5,7^ and Alessandra Aloisi.^*,5^

^1^Mathematics and Physics “E. De Giorgi” Department, University of Salento, Via Arnesano, Lecce, 73100, Italy

^2^Center for Biomolecular Nanotechnologies (CBN), Istituto Italiano di Tecnologia (IIT), Via Barsanti, Arnesano, 73010 Lecce, Italy

^3^Department of Pharmacy, University of Pisa, Via Bonanno Pisano, Pisa, 56126, Italy

^4^MARine PHARMA Center, Centro interdipartimentale di Farmacologia marina, University of Pisa, Via Bonanno Pisano, Pisa, 56126, Italy

^5^Institute for Microelectronics and Microsystems (IMM), CNR, Via Monteroni, Lecce, 73100, Italy

^6^Oligonucleotides Laboratory, Institute of Clinical Physiology (IFC), CNR, Via Moruzzi, Pisa, 56124, Italy

^7^ISUFI, University of Salento, Via Monteroni, Lecce, 73100, Italy

^a^Current affiliation: Department of Neuroscience and Brain Technologies (NBT), Istituto Italiano di Tecnologia (IIT), Via Morego, Genova, 16163, Italy

^b^Current affiliation: Institute for Microelectronics and Microsystems (IMM), CNR, Via Monteroni, Lecce, 73100, Italy

Experimental

S1. TEM analysis

TEM analysis was performed with a JEOL JEM-1011 transmission electron microscope at 100 kV operating voltage, equipped with a 7.1 megapixel CCD camera (Orius SC1000, Gatan, Pleasanton, CA) and TEM image analysis was achieved with Gatan Digital Micrograph™ (DM) software. By TEM analysis, ultra-microtome- based trans-sectional imaging was performed. Particles were fixed with glutaraldehyde in UPW 2% (*v/v*) for 20 minutes, followed by further fixation in OsO_4_ 1% (*v/v*) in UPW for 1h; triple washings were performed in UPW for 5 minutes after each step. Samples were successively dehydrated by 50, 70, 90, 95 % ethanol for 5 minutes each and 100% absolute ethanol for 5 minutes (thrice). Samples were then embedded in epoxy resin (Sigma Aldrich) and allowed to polymerize at 70°C for 48 h. Ultra-thin sections (70 nm thick) were cut by using ultra-microtome (PTPC PowerTome, RMC-Boeckeler Instruments, Tucson, US) equipped with a wet diamond knife and the sections were picked up onto 400 mesh copper TEM grids.

S2. AFM analysis

AFM images were recorded in air at RT using a Nanoscope VI Multimode Scanning Probe workstation (Bruker, Germany) and J scanner. Samples were imaged in tapping mode, with Antimony (n) doped Silicon probes (RFESPA-75, Bruker), nominal frequency 75 kHz, nominal spring constant of 3 N/m, nominal radius of 8 nm. Hence, consecutive shots were monitored before collecting images at field size~~s~~ of 1 µm^2^, with 1024×1024 pixel resolution, and scan rate set at 0.996 Hz. Image processing and analysis were carried out using NanoScope TM version 7.30 software (Veeco/Digital Instruments, Santa Barbara, CA).

S3. Confocal analysis

Adherent MeWo cells, cultured on cover slides, at 60 % confluence were exposed for 24 h to 50 µg/ml of oligo-spd-SNPs (corresponding to a ~~5~~ 100 nM oligo). After the treatment, cells were washed thrice with PBS, fixed with 4% (*w/v*) of paraformaldehyde and rinsed thrice with PBS; nuclei were stained using diamidino-2-phenylindole (DAPI); oligo-spd-SNPs were imaged availing of oligo 6-FAM label. Images were recorded on Leica TCS SP8 STED CW system.

S4. SEM

A Zeiss Sigma VP scanning electron microscope, equipped with a Field Emission Gun and a high resolution SEM Gemini column, was used for the morphological analyses. The measurements were carried out at 5 kV accelerating voltage, which represents a good compromise between enhancing the surface morphology and minimizing the charging effects. The Everhart Thornley Secondary electron detector was used for the acquisition of the images.

S5. DLS

The average hydrodynamic diameter measurements were performed on Zetasizer Nano ZSP instrument (ZEN5600, Malvern, United Kingdom) equipped with a 10 mW He–Ne laser operating at 633 nm, fixed scattering angle of 173°. DLS technique has also been used to perform particle dissolution test in PBS solution at pH 5.5, 6.5 and 7.2 after 5 days of incubation.

S6. Spectrophotometric analysis

The spectrophotometric analysis was performed on BioTek’s Synergy Mx Monochromator-based Microplate Reader (BioTeK, USA), controlled by [Gen5](https://www.biotek.com/products/software-robotics-software/gen5-microplate-reader-and-imager-software/) Software for data collection and analysis. Fluorescence signal determinations were made using a high energy xenon flash lamp and a double grating monochromator (top/bottom).

S7. FTIR

IR-spectra were acquired using a JASCO FT/IR6800 (JASCO, Japan) spectrometer. The samples were drop-casted onto disposable Real Crystal IR sample cards (KBr window with an aperture of 15 mm) and measured under vacuum after complete drying. Spectra were acquired in the range of 4000-400 cm^-1^ (resolution of 4 cm^-1^) at room temperature, with the accumulation of 32 repeated scans. Spectral manipulations were performed using the spectral analysis software from Jasco.

S8. ICP

ICP-OES (720 ICP-OES, Agilent Technologies, Santa Clara, US) was performed in order to value Si content in 1 mg of SPs after incubation in 3.2 mM spermidine or in UPW. The mix was stirred at 700 rpm, for 15 h at room temperature and then precipitated at 13000 rpm for 30 minutes. Both pellet and supernatant were lyophilized, and digested with concentrated nitric acid, for ICP evaluation.


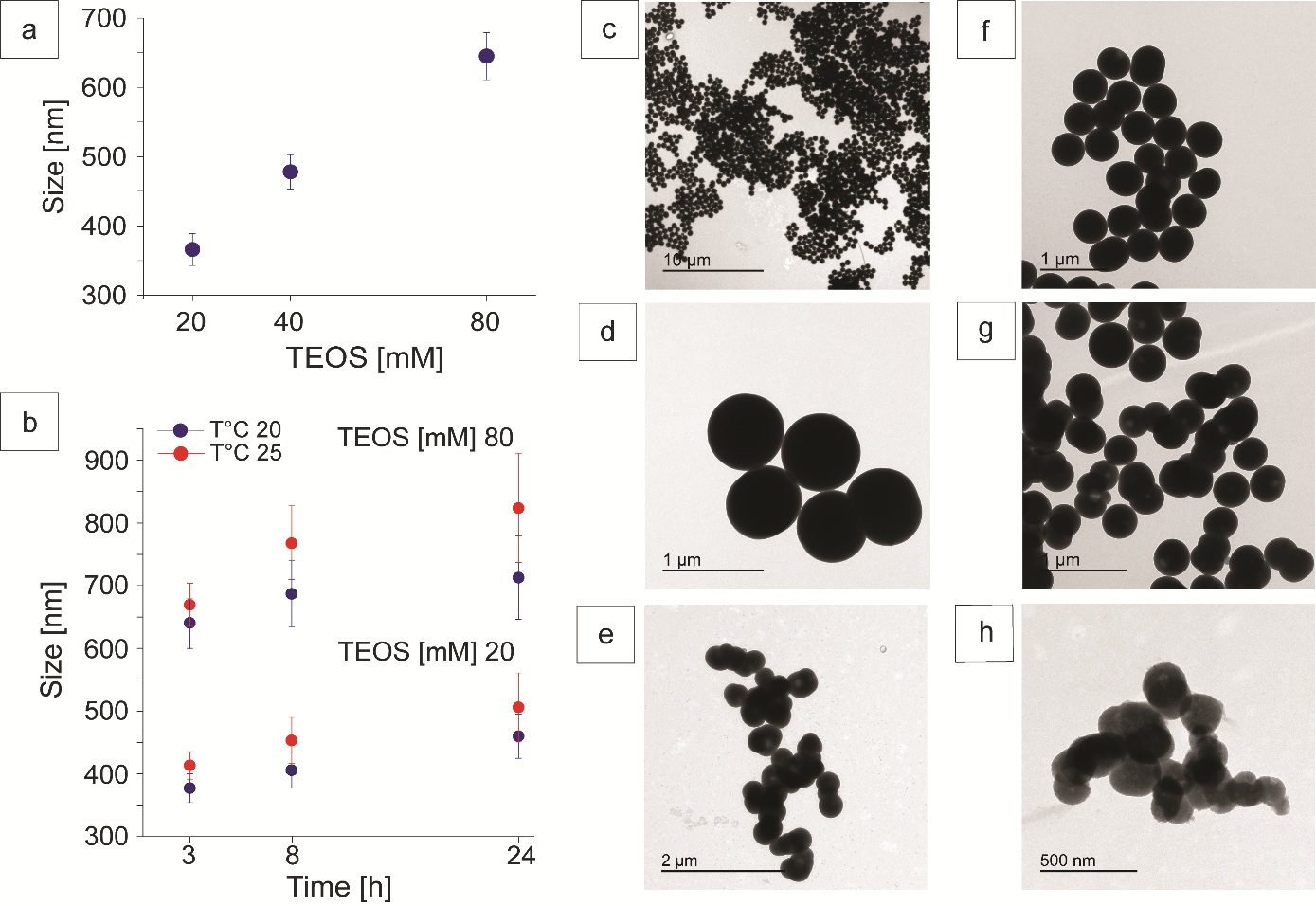


| **Ethanol 15% (v/v) [ml]** | **TEOS**  **[mM]** | **Spd**  **[mM]** | **Spd/TEOS** | **Time**  **[h]** | **Temperature**  **[°C]** | **Size**  **[nm]** | **TEM**  **Image** |
| --- | --- | --- | --- | --- | --- | --- | --- |
| 1 | 20 | 0.16 | 0.008 | 3 | 20 | 177 ± 25 | h |
| 1 | 80 | 1.6 | 0.02 | 3 | 25 | 823 ± 92 | d |
| 1 | 80 | 1.6 | 0.02 | 3 | 20 | 643 ± 36 | g |
| 1 | 20 | 0.8 | 0.04 | 3 | 20 | 350 ± 38 | e |
| 1 | 40 | 1.6 | 0.04 | 3 | 25 | 496 ± 38 | c |
| 1 | 20 | 1.6 | 0.08 | 3 | 20 | 377 ± 23 | f |

**Figure S1**. Effect of reaction conditions on SP size. (a) SP average diameter at different TEOS concentrations (fixed spermidine concentration, 1.6 mM). Particle size increases with theTEOS increasing. (b) Effect of reaction time and temperature on SP size synthesized in the presence of 80 mM or 20 mM TEOS (fixed spermidine concentration, 1.6 mM). At higher temperature and longer time, SPs present a larger size and larger polydispersity. TEM images of representativeSPs prepared varying reaction conditions: (c) 1.6 mM spermidine and 40 mM TEOS, at 25°C for 3 h; (d) 1.6 mM spermidine and 80 mM TEOS, at 25°C for 3 h; (e) 0.8 mM spermidine and 20 mM TEOS, at 20°C for 3 h; (f) 1.6 mM spermidine and 20 mM TEOS, at 20°C for 3h; (g) 1.6 mM spermidine and 80 mM TEOS, at 20°C for 3 h; (h) 0.16 mM spermidine and 20 mM TEOS, at 20°C for 3 h. Relative average diameters of SPs are reported in the table in function of increasing Spd/TEOS ratio. SP average diameter was determined analyzing the diameter of about a 50-100 particles/sample from TEM micrographs.


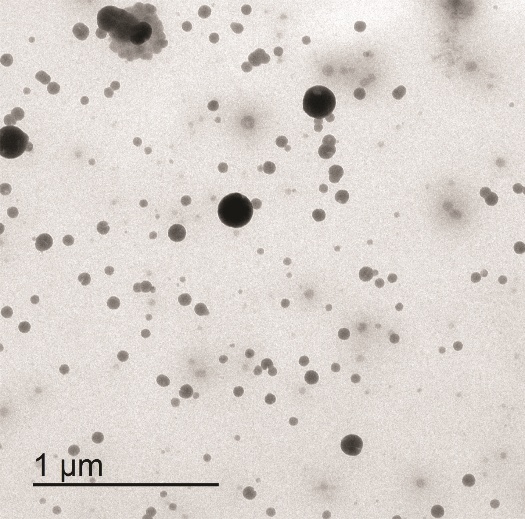


**Figure S2.** TEM image of SiO_2_-based nanostructures produced during SP emptying step. Inorganic component dissolves from SP matrix and re-precipitates by the competitive effect of supplementary free spermidine added in solution, forming novel hybrid *Pac-Man*-like structures.


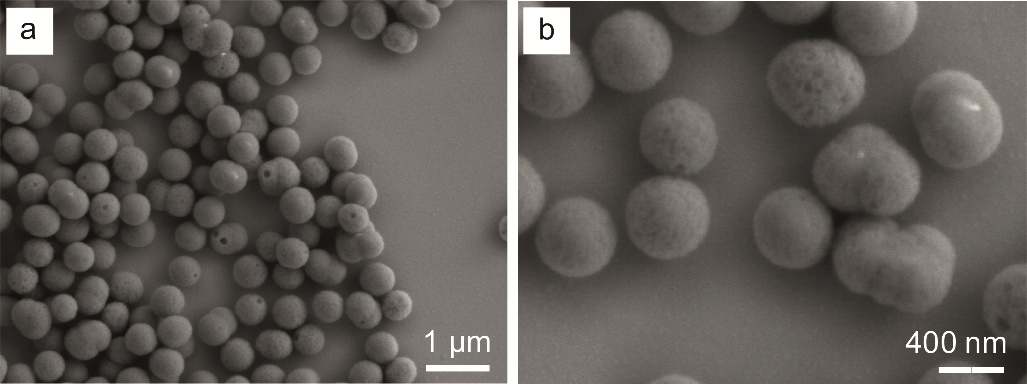


**Figure S3.** SEM images of SPs after stirring step in UPW (15 h). SPs present a rough surface. Compared with 15 h stirring step with additional free spermidine (**Figure 5** in the manuscript), a reduced silica dissolution is evidenced by limited pitting effect.


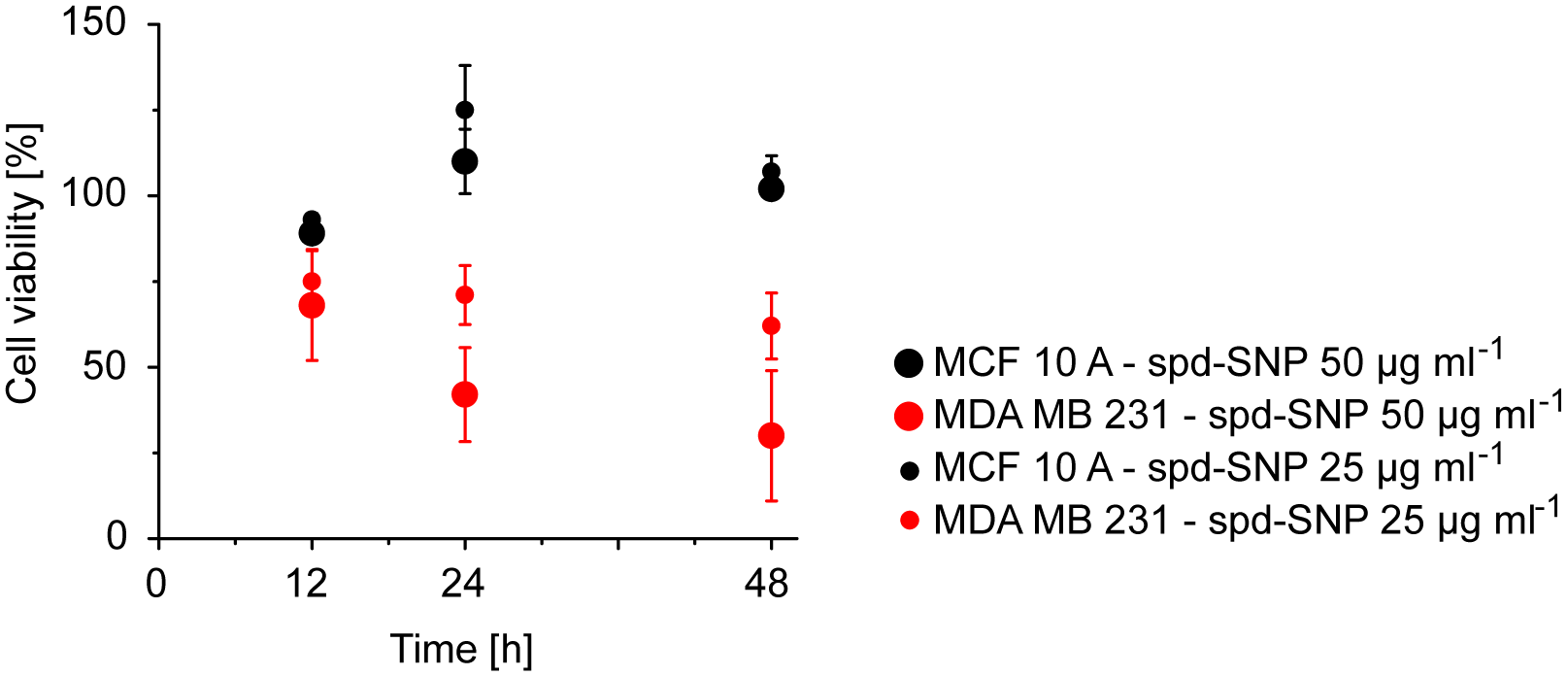


**Figure S4**. Cytotoxicity of 25 or 50 μg ml^-1^ spd-SNPs to breast cancer cell line (MDA-MB-231) over non-tumorigenic breast epithelial cell line (MCF 10A). MDA-MB-231 and MCF 10A were treated with 25 or 50 μg ml^-1^ spd-SNPs for 24 h and cell viability was assessed 12, 24 and 48 h later, by ATPlite assay.

Spd-SNPs are selectively toxic to MDA-MB-231 breast tumor cells. After 48 h treatment with 25 or 50 μg ml^-1^ spd-SNPs, the percentage of MDA-MB-231 viable cells decreased to 62 ± 9 % and to 30 ± 19 %, respectively. Conversely, spd-SNP do not affect MCF 10 A viability. Data are expressed as mean value ± SD (n=3).


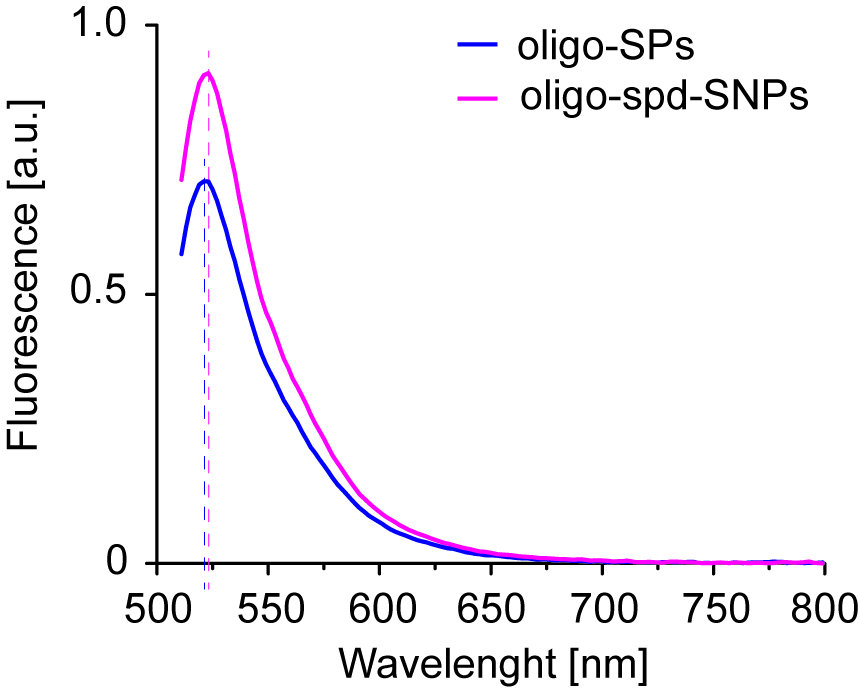


**Figure S5**. Fluorescence spectrum (Fixed Excitation: 495/9.0 nm, Emission Start: 505/9.0 nm) of FAM-labeled oligonucleotide (450 nM) loaded in SPs (200 μg) or spd-SNPs (200 μg). Spd-SNP fluorescence spectrum presents a slight bathochromic shift from 521 to 523 nm.

| **Particle Type** | **Time 0** |  | **Time 5 days** |  |
| --- | --- | --- | --- | --- |
|  | **Size**  **[nm]** | **pH 5.5**  **Size[nm]** | **pH 6.5**  **Size[nm]** | **pH 7.2**  **Size[nm]** |
| SPs | 520.0 ± 38.2  (PDI, 0.182) | 477 ± 31.5  (PDI, 0.730) | 415.8 ± 29.1  (PDI, 0.781) | 393.5 ± 11.4  (PDI, 0.693) |
| spd-SNPs | 352.4 ± 38.4  (PDI, 0.278) | 312.1 ± 22.4  (PDI, 0.820) | 253.4 ± 7.1  (PDI, 0.875) | 198.3 ± 13.4  (PDI, 0.780) |

**Table S1.** DLS particle size measurements of SPs and spd-SNPs at time 0 and after 5 days incubation in PBS at pH 5.5, 6.5 and 7.2.


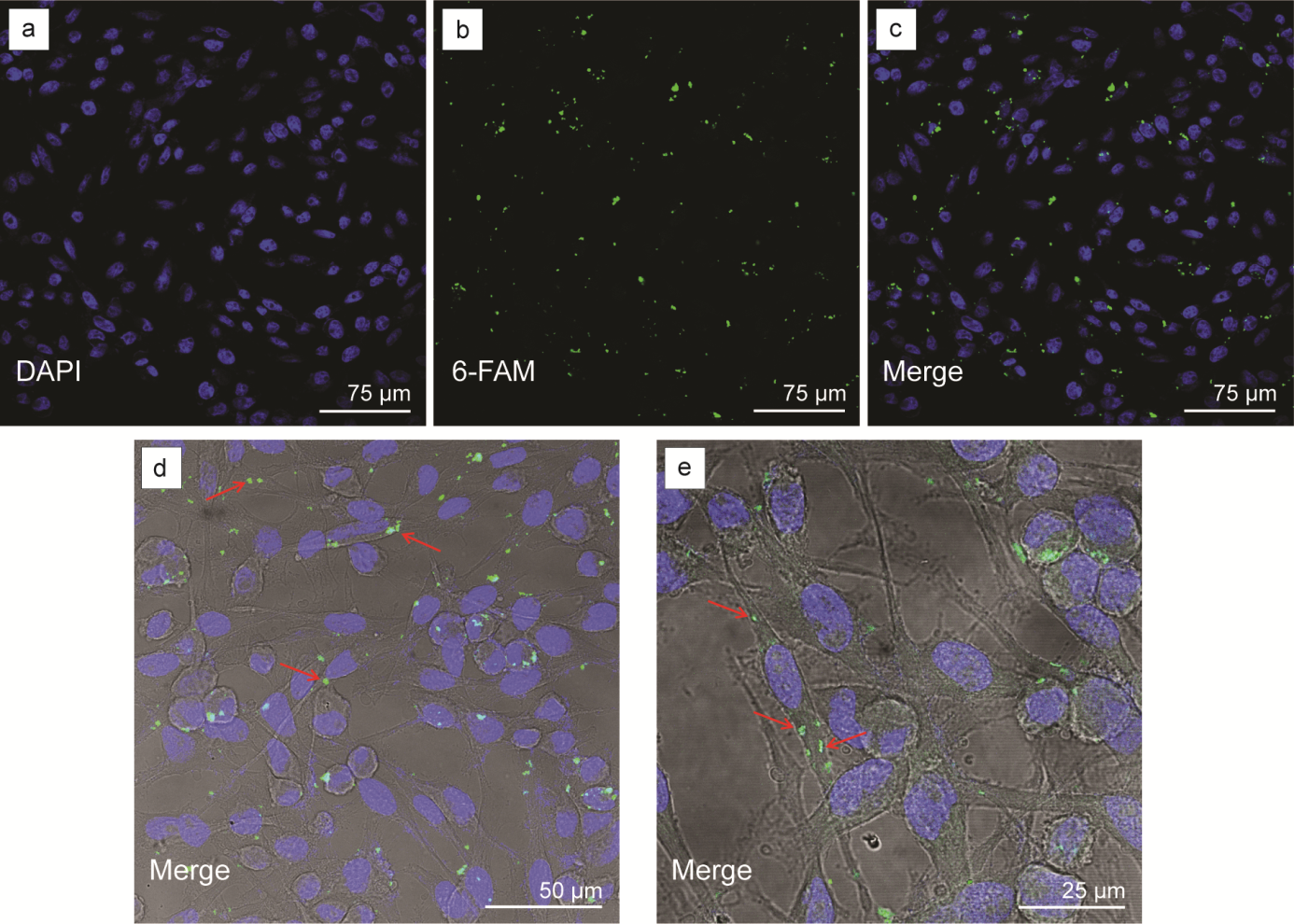


**Figure S6.** Cellular uptake of oligonucleotide loaded spd-SNPs. Separated and merged channels of MeWo cell confocal microscopy images recorded on Leica SP8 STED CW. Green florescence of (6-FAM)-labeled oligonucleotide loaded on spd-SNPs; blue florescenceof nuclei stained with Hoechst 33258. (a, b, c) DAPI, FITC, channel overlay (40x/1.30 oil). Higher magnification (obj 63x/1.40 oil, 100x/1.40 oil) and transmission channel overlay (d, e) showing particle distribution within cell cytosol, indicated by red arrows. Images were taken after 24 h incubation.


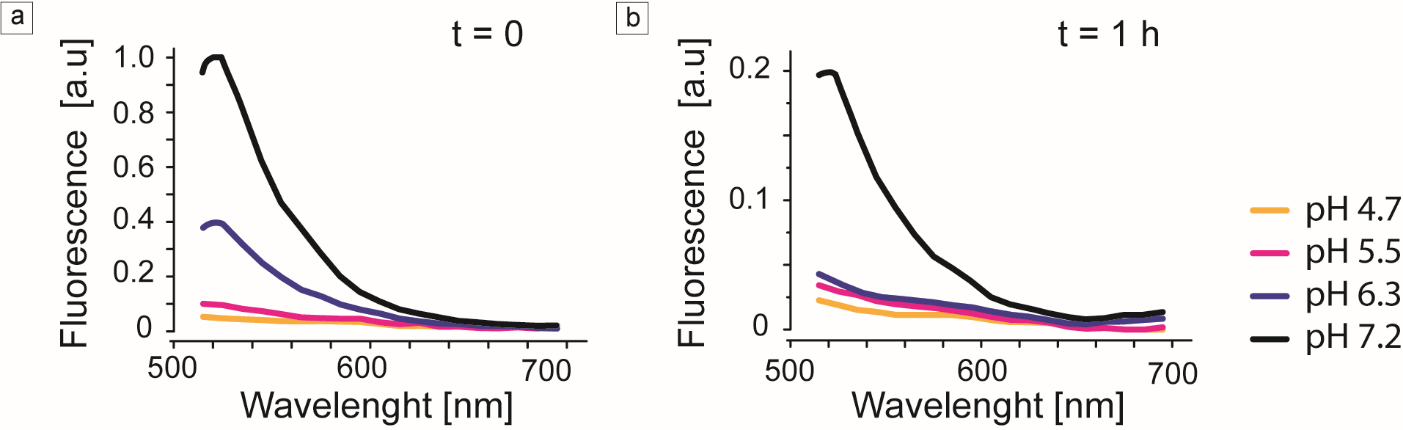


**Figure S7.** Time-dependent PL spectra, (a) t=0 (b) t=1 hour, of (6-FAM)-labeleld oligonucleotide (siR-scramble) release from spd-SNP in citrate phosphate buffer at pH 4.7, 5.5, 6.3, and 7.2, which respectively simulate the lysosome, late and early endosome and cytosolic enviroment. Once siR-scramble@spd-SNP complex is achieved as described in Experimental section - 'Oligonucleotide loading', PL spectrum was measured before and after 1 hour release in the microplate reader set at 37°C (Fixed Excitation: 495/9.0 nm, Emission Start: 505/9.0 nm). Remarkably, the higher release trend at acidic pH 6.3 should allow the successive oligo endosome escape [Juliano, R.L, 2018], if the carrier could not; as well, release is permitted in the cytosol, at pH 7.2.

References

Juliano, R.L. Intracellular Trafficking and Endosomal Release of Oligonucleotides: What We Know and What We Don’t. Nucleic Acid Ther. 28, 166-177 (2018)
